# The association of *APE1 *−656T > G and 1349 T > G polymorphisms and cancer risk: a meta-analysis based on 37 case-control studies

**DOI:** 10.1186/1471-2407-11-521

**Published:** 2011-12-18

**Authors:** Bin Zhou, Hailin Shan, Ying Su, Kai Xia, Xiaxia Shao, Weidong Mao, Qing Shao

**Affiliations:** 1 Department of General Surgery, Jiangyin People's Hospital, South-East University, Jiangyin, China; 2 Department of Oncology, Jiangyin People's Hospital, South-East University, Jiangyin, China; 3 Department of General Surgery, Jiangyin People's Hospital, 163 Shoushan Road, Jiangyin 214400, China

**Keywords:** c*APE1*, Single nucleotide polymorphism, Cancer risk, Meta-analysis

## Abstract

**Background:**

APE1 (apurinic/apyrimidinic endonuclease 1) is an important DNA repair protein in the base excision repair pathway. Polymorphisms in *APE1 *have been implicated in susceptibility to cancer; however, results from the published studies remained inconclusive. The objective of this study was to conduct a meta-analysis investigating the association between polymorphisms in *APE1 *and the risk for cancer.

**Methods:**

The PubMed and Embase databases were searched for case-control studies published up to June, 2011 that investigated *APE1 *polymorphisms and cancer risk. Odds ratios (ORs) and 95% confidence intervals (CIs) were used to assess the strength of the associations.

**Results:**

Two polymorphisms (−656 T > G, rs1760944 and 1349 T > G, rs1130409) in 37 case-control studies including 15, 544 cancer cases and 21, 109 controls were analyzed. Overall, variant genotypes (GG and TG/GG) of −656 T > G polymorphism were associated with significantly decreased cancer risk in homozygote comparison (OR = 0.81, 95%CI: 0.67-0.97), dominant model comparison (OR = 0.89, 95%CI: 0.81-0.97) and recessive model comparison (OR = 0.90, 95%CI: 0.82-0.98), whereas the 1349 T > G polymorphism had no effects on overall cancer risk. In the stratified analyses for −656 T > G polymorphism, there was a significantly decreased risk of lung cancer and among Asian populations.

**Conclusions:**

Although some modest bias could not be eliminated, the meta-analysis suggests that *APE1 −*656 T > G polymorphism has a possible protective effect on cancer risk particularly among Asian populations whereas 1349 T > G polymorphism does not contribute to the development of cancer.

## Background

Cancer is a multifactor disease caused by complex interactions between environmental and genetic factors [1]. The incidence of different cancer varies widely in different populations which may be largely attributed to lifestyle and genetic background [2]. Environmental factors such as smoking and exposure to carcinogens lead to direct damage to DNA [3]. To a certain extent, these damages can be repaired by endogenous DNA repair systems [4]. However, genetic variations in these systems may result in reduced repair capability and the defect allow DNA damage to accumulate which leads to permanent mutations in the genome and contributes to carcinogenesis.

Among DNA repair systems, base excision repair (BER) pathway is responsible for repairing small lesions such as oxidative damage, alkylation, or methylation [5]. AP Endonuclease 1 (APE1, also known as APE, APEX, HAP1, and REF-1) is a multifunctional protein and plays a central role in the BER pathway through hydrolyzing the phosphodiester backbone immediately 5' to the AP site [5,6]. APE1 can act as a 3'-phosphodiesterase to initiate repair of DNA single strand breaks, which are produced either directly by reactive oxygen species or indirectly through the enzymatic removal of damaged bases [7,8]. APE1 is also known as a transcriptional coactivator for numerous transcription factors involved in cancer development [9] and is considered as a promising tool for anticancer therapy [10].

The human *APE1 *is located on chromosome 14q11.2-q12 and consists of five Exons spanning 2.21 kb [11]. Till now, many epidemiological studies suggested genetic variations in *APE1 *may confer individuals' susceptibility to cancer. A total of 18 single nucleotide polymorphisms (SNPs) in *APE1 *have been identified [11], of which, two functional SNPs (−656 T > G in the promoter region, rs1760944 and 1349 T > G in the fifth Exon, rs1130409) have been wildly investigated. In lung cancer, Lu et al. and Lo et al. reported that −656 T > G polymorphism influenced the transcriptional activity of *APE1 *and contributed to lung cancer susceptibility [12,13]. Although one study in bladder cancer supported the association [14], it has not been replicated in some others [15-17]. As to 1349 T > G polymorphism, functional studies suggested that the G allele may have altered endonuclease and DNA-binding activity, reduced ability to communicate with other BER proteins and decreased capacity to repair DNA oxidative damage [18,19]. Recently, numerous studies investigated the influence of 1349 T > G polymorphism on cancer risk; however, the results of these studies remain inconclusive.

To reveal a small effect of the polymorphisms on cancer risk, a single study might be underpowered, particularly for studies with relatively small sample size. Meta-analysis is a statistical technique for combining results from different studies to produce a single estimate of the major effect with enhanced precision. It is considered a powerful tool to summarize inconclusive results from different studies. To clarify the effect of the *APE1 *-656 T > G and 1349 T > G polymorphisms on cancer risk; we conducted a meta-analysis of all eligible case-control studies that have been published. Before analysis, we tested whether these two SNPs were in linkage disequilibrium with each other in Europeans and Asians, and the data from HapMap database suggested the two SNPs were not correlated (*D' *= 0.335, r^2 ^= 0.071 for Europeans and *D' *= 0.409, r^2 ^= 0.071 for Asians).

## Methods

### Identification of eligible studies

A literature search of the PubMed and EMBASE databases (updated to June, 2011) was conducted using combinations of the following terms: "APE1", "APEX", "HAP1", "REF-1", "polymorphism", and "cancer". The search was limited to English language papers. All studies that evaluated the association between polymorphisms of *APE1 *and cancer risk were retrieved, and their bibliographies were checked for other relevant publications. Studies that were included in the meta-analysis had to meet all of the following criteria: 1) evaluate the *APE1 −*656T > G and 1349T > G polymorphisms and cancer risk, 2) use a case-control design, 3) contain available genotypes frequency for estimating an odds ratio (OR) with a 95% confidence intervals (CIs), 4) genotype distributions in control consistent with Hardy-Weinberg equilibrium (HWE). Accordingly, abstracts and reviews studies that that did not report genotype frequency were excluded. When more than one of the same patient population was included in several publications, only the most recent or complete study was used in this meta-analysis.

### Data extraction

Information was carefully extracted from all eligible publications independently by two of the authors according to the selection criteria. In case of disagreement, a third reviewer assessed the articles. The following items were collected: author name, year of publication, country of origin, ethnicity, source of the controls, genotyping method, cancer type, total number of cases and controls, and genotype distributions in cases and controls.

### Statistical analysis

The strength of associations between *APE1 *polymorphisms and cancer risk were measured by ORs with 95%CIs. We examined the association between the *APE1 −*656 T > G and 1349 T > G polymorphisms and cancer risk in homozygote comparison (GG vs. TT), heterozygote comparison (TG vs. TT), the dominant genetic model (TG/GG vs. TT), and the recessive genetic model (GG vs. TT/TG). Stratified analyses were also carried out by ethnicity, cancer type and source of controls.

Heterogeneity assumption was evaluated with a chi-square-based Q-test. If the *P *value is greater than 0.05 of the Q-test, which indicates a lack of heterogeneity among studies, the summary OR estimate of each study was calculated by a fixed effects model (the Mantel-Haenszel method) [20]; otherwise, the random-effects model (the DerSimoniane and Laird method) [21] was performed. Sensitivity analyses were also performed to assess the stability of the results. Funnel plots and Egger's linear regression test were used to provide diagnosis of the potential publication bias. All statistical analysis were done with the Stata software (version 11.0; StataCorp LP, College Station, TX), using two-sided *P *values.

## Results

### Study selection and characteristics in the meta-analysis

A total of 37 eligible studies from 32 articles met the inclusion criteria [12-17,22-47]. In total, 5,139 cases and 5,201 controls from 8 studies for −656 T > G polymorphism [12-17], and 14,222 cases and 19,746 controls from 35 studies for 1349 T > G polymorphism were included in the pooled analyses [12-14,16,17,22-47]. The characteristics of selected studies are presented in Table 1. The distribution of genotypes in the controls of all studies was consistent with HWE. Of the 8 studies for −656 T > G polymorphism, 3 investigated lung cancer and 2 investigated bladder cancer, and the other 3 articles investigated individually brain cancer, renal cell cancer and colorectal adenoma. Six of these studies were conducted in Asian populations and 2 in European populations. Among the 35 studies for 1349 T > G polymorphism, there were 7 bladder cancer studies, 4 breast cancer studies, 4 colorectal cancer studies, 2 head and neck cancer studies, 9 lung cancer studies and 9 others studies including oesophageal cancer, pancreatic cancer, leukaemia, prostate cancer, biliary tract cancer, thyroid cancer, renal cell cancer and gastric cancer. Ten of these studies were from Asians, 23 were from Europeans and 2 were studies of mixed populations.

**Table 1:** Characteristics of populations and cancer types of the studies included in the meta-analysis

Study	Country	Ethnicity	Cancer type	Sample size case/control	Source of controls	Genotyping method	Polymorphisms
Misra 2003 ^20^	Finland	European	Lung cancer	310/302	Population	TaqMan	1349 T > G

Popanda 2004 ^21^	Germany	European	Lung cancer	459/457	Hospital	Rapid capillary PCR	1349 T > G

Hao 2004 ^22^	China	Asian	Oesophageal cancer	409/478	Population	PCR-RFLP	1349 T > G

Ito 2004 ^23^	Japan	Asian	Lung cancer	178/449	Hospital	PCR-RFLP	1349 T > G

Shen 2005 ^24^	China	Asian	Lung cancer	117/113	Population	TaqMan	1349 T > G

Moreno 2006 ^25^	Spain	European	Colorectal cancer	359/312	Hospital	Arrayed primer extension	1349 T > G

Jiao 2006 ^26^	USA	European	Pancreatic cancer	367/330	Hospital	Allele-specific PCR	1349 T > G

Terry 2006 ^27^	USA	European	Bladder cancer	229/207	Hospital	MALDI-TOF	1349 T > G

Wu 2006 ^28^	USA	European	Bladder cancer	596/590	Hospital	TaqMan	1349 T > G

Zhang 2006 ^29^	USA	European	Breast cancer	1529/1207	Population	TaqMan	1349 T > G

Matullo 2006 ^30^	Multi-country	European	Breast cancer	124/1094	Population	TaqMan	1349 T > G

			Lung cancer	116/1094	Population	TaqMan	1349 T > G
			
			Head and neck cancer	82/1094	Population	TaqMan	1349 T > G
			
			Leukemia	169/1094	Population	TaqMan	1349 T > G
			
Chen 2006 ^31^	USA	Mixed	Prostate cancer	351/329	Hospital	PCR-RFLP	1349 T > G

Li 2007 ^32^	USA	European	Head and neck cancer	830/854	Hospital	PCR-RFLP	1349 T > G

Berndt 2007 ^14^	USA	European	Colorectal cancer	767/773	Population	TaqMan	1349 T > G, −656 T > G

Figueroa 2007 ^15^	Spain	European	Bladder cancer	1150/1149	Hospital	TaqMan	1349 T > G, −656 T > G

Andrew 2008 ^33^	USA/Italy	European	Bladder cancer	911/1165	Population	SNP mass-tagging system	1349 T > G

Pardini 2008 ^34^	Czech	European	Colorectal cancer	531/530	Hospital	TaqMan	1349 T > G

Mitra 2008 ^35^	India	European	Bladder cancer	150/225	Population	PCR-RFLP	1349 T > G

Tse 2008 ^36^	USA	European	Colorectal cancer	311/454	Hospital	TaqMan	1349 T > G

Huang 2008 ^37^	China	Asian	Biliary tract cancer	409/783	Population	TaqMan	1349 T > G

Chiang 2008 ^38^	China	Asian	Thyroid cancer	283/469	Hospital	TaqMan	1349 T > G

Smith 2008 ^39^	USA	Asian	Breast cancer	372/480	Hospital	Arrayed primer extension	1349 T > G

Gangwar 2009 ^40^	India	European	Bladder cancer	206/250	Hospital	TaqMan	1349 T > G

Agachan 2009 ^41^	Turkey	European	Lung cancer	98/67	Hospital	PCR-RFLP	1349 T > G

Lo 2009 ^11^	China	Asian	Lung cancer	729/722	Hospital	Arrayed primer extension	1349 T > G, −656 T > G

Lu 2009 ^10^	China	Asian	Lung cancer	500/517	Population	Illumina	1349 T > G, −656 T > G

			Lung cancer	572/547	Hospital	SNPscan	−656 T > G
			
Wang 2010 ^12^	China	Asian	Bladder cancer	234/253	Hospital	PCR-RFLP	1349 T > G, -656 T > G

Deng 2010 ^42^	China	Asian	Lung cancer	315/314	Population	RT-PCR	1349 T > G

Palli 2010 ^43^	Italy	European	Gastric cancer	298/546	Population	TaqMan	1349 T > G

Jelonek 2010 ^44^	Poland	European	Colorectal cancer	113/153	Hospital	PCR-RFLP	1349 T > G

			Breast cancer	91/412	Hospital	PCR-RFLP	1349 T > G
			
Zhou 2011 ^13^	China	Asian	Brain cancer	750/816	Hospital	MassARRAY	−656 T > G

Cao 2011 ^45^	China	Asian	Renal cell carcinoma	612/632	Hospital	TaqMan	1349 T > G, -656 T > G

*RFLP*, restriction fragment length polymorphism; PCR, polymerase chain reaction; MALDI-TOF, matrix assisted laser desorption/ionization time-of-flight; SNP, single nucleotide polymorphism; C, cytosine; T, thymine; PCR-CTPP, polymerase chain reaction with confronting two-pair primers.

### The −656 T > G polymorphism

The frequency of the G allele varied widely across the 8 studies, ranging from 0.44 to 0.64. The average frequency of the G allele in Asian populations was 0.45, which was lower than that in European populations (0.62). The difference was statistical significant (*P *< 0.001).

**Figure 1:**
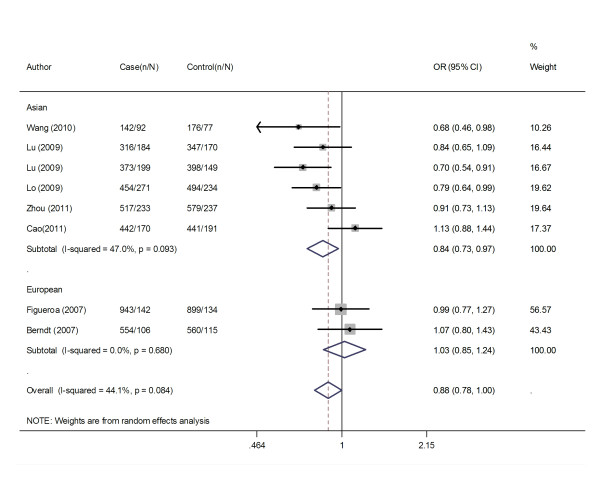
**Meta-analysis with a random-effects model for the association between cancer risk and the *APE1 *−656 T > G polymorphism stratified by ethnicity (TG/GG *vs*. TT)**. OR, odds ratio; CI, confidence interval; I-squared, measure to quantify the degree of heterogeneity in meta-analyses.

**Figure 2:**
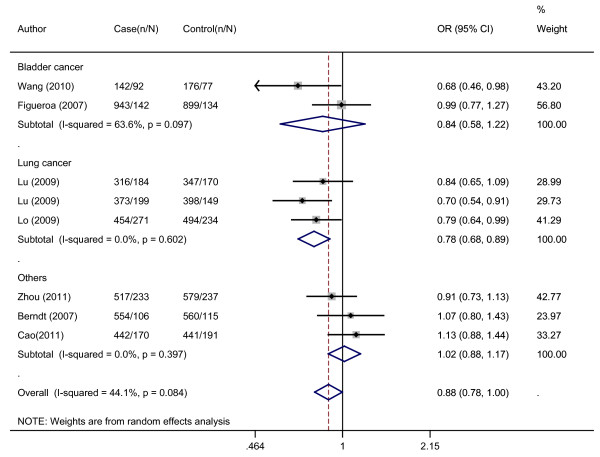
**Meta-analysis with a random-effects model for the association between cancer risk and the *APE1 *-656 T > G polymorphism stratified by cancer types (TG/GG *vs*. TT)**. OR, odds ratio; CI, confidence interval; I-squared, measure to quantify the degree of heterogeneity in meta-analyses.

Overall, there was evidence of an association between decreased cancer risk and the variant genotypes in different genetic models when all the eligible studies were pooled into the meta-analysis. As show in Table 2, compared with the wild-type homozygote genotype, the homozygote variant genotype GG was associated with a statistically significant decreased risk of all types of cancer (OR = 0.81, 95%CI = 0.67-0.97). Besides, significant main effects were also observed both in dominant and recessive models (OR = 0.89, 95%CI = 0.81-0.97 and OR = 0.90, 95%CI = 0.82-0.98). In the stratified analysis by populations, as shown in Table 2 and Figure 1, the effect was remain in studies of Asian populations (homozygote comparison: OR = 0.75, 95%CI = 0.65-0.86; heterozygote comparison: OR = 0.89, 95%CI = 0.80-0.99; dominant model: OR = 0.85, 95%CI = 0.77-0.94 and recessive model: OR = 0.81, 95%CI = 0.71-0.91). In the stratified analysis by cancer type, as shown in Table 2 and Figure 2, the effect was remain in lung cancer studies (homozygote comparison: OR = 0.65, 95%CI = 0.54-0.79; heterozygote comparison: OR = 0.83, 95%CI = 0.72-0.97; dominant model: OR = 0.78, 95%CI = 0.68-0.89 and recessive model: OR = 0.72, 95%CI = 0.61-0.86). Further subgroup analyses were not performed because of limited data for this polymorphism.

**Table 2:** Meta-analysis of the *APE1 *−656 T > G polymorphism and cancer risk

Variables	n *	GG vs. TT		TG vs. TT		GG/TG vs. TT		GG vs. TT/TG	
	
		OR (95% CI) ^†^	*P *^‡^	OR (95% CI) ^†^	*P *^‡^	OR (95% CI) ^†^	*P *^‡^	OR (95% CI) ^†^	*P *^‡^
Total	8	0.81 (0.67-0.97)	0.015	0.92 (0.83-1.01)	0.340	0.89 (0.81-0.97)	0.084	0.90 (0.82-0.98)	0.059

Ethnicities									

Asian	6	0.75 (0.65-0.86)	0.057	0.89 (0.80-0.99)	0.255	0.85 (0.77-0.94)	0.093	0.81(0.71-0.91)	0.252

European	2	1.04 (0.85-1.28)	0.703	1.01 (0.83-1.24)	0.692	1.03 (0.85-1.24)	0.680	1.03 (0.90-1.18)	0.891

Cancer types									

Lung cancer	3	0.65 (0.54-0.79)	0.857	0.83 (0.72-0.97)	0.469	0.78 (0.68-0.89)	0.602	0.72 (0.61-0.86)	0.799

Bladder cancer	2	0.88 (0.70-1.12)	0.045	0.89 (0.72-1.11)	0.583	0.88 (0.71-1.08)	0.097	0.97 (0.82-1.14)	0.086

Others	3	1.00 (0.83-1.19)	0.276	1.03 (0.88-1.19)	0.340	1.02 (0.88-1.15)	0.397	0.98 (0.85-1.13)	0.437

* Number of comparisons

^† ^Random-effects model was used when P value for heterogeneity test < 0.05; otherwise, fix-effects model was used

^‡ ^P value of Q-test for heterogeneity test

Heterogeneity was observed in homozygote comparison (*P *_heterogeneity _= 0.015) for the main effects. Therefore, we conducted sensitivity analyses to locate the source of heterogeneity, and the results suggested that two studies [16,47] were the main origin of the heterogeneity. The heterogeneity was effectively removed after exclusion of these studies (*P *_heterogeneity _= 0.123, 0.355 and 0.163, respectively for homozygote comparison, dominant and recessive model). The results were not materially altered after exclusion of these studies, suggesting the results of this meta-analysis were stable.

**Figure 3:**
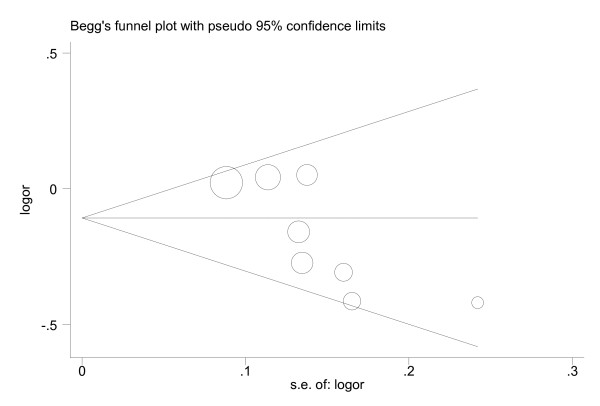
**Begg's funnel plot of publication bias test (−656 T > G; GG *vs*. TT/TG)**. Each point represents a separate study for the indicated association. Log (OR), natural logarithm of OR; Horizontal line, mean effect size.

As shown in Figure 3, the shape of the funnel plots seemed asymmetrical in the recessive model, suggesting the presence of publication bias. The Egger's test showed obvious evidence of publication bias (t = −3.01, *P *= 0.024, recessive model). To adjust for this bias, a trim-and-fill method developed by Duval and Tweedie [48] was conducted, the adjusted estimates obtained by using random-effects model was OR of 0.87 (0.76-0.99). Meta-analysis with or without the trim-and-fill method did not draw different conclusions, indicating that our results were statistically robust.

### The 1349 T > G polymorphism

The frequency of the G allele varied widely across the 39 studies, ranging from 0.20 to 0.52. However, no significant difference in the frequency distribution of the G allele between Asian and European populations were observed (frequencies of G allele were 0.42 and 0.44 in Asian and European populations, respectively; *P *= 0.514).

As shown in Table 3, the results did not suggest any statistical evidence of an association between the 1349 T > G polymorphism and overall cancer risk (Table 3). In the subgroup analysis by ethnicities, source of controls and cancer types, still no significant association was observed (Table 3). No publication bias was detected by either the funnel plot or the Egger test (GG vs. TT: t = 0.33, *P *= 0.745; TG vs.TT: t = 1.22, *P *= 0.233; TG/GG vs. TT: t = 1.29, *P *= 0.244; GG vs. TT/TG: t = 0.02, *P *= 0.985).

**Table 3:** Meta-analysis of the *APE1 *1349 T > G polymorphism and cancer risk

Variables	n *	GG vs. TT		TG vs. TT		GG/TG vs. TT		GG vs. TT/TG	
	
		OR (95% CI) ^†^	*P *^‡^	OR (95% CI) ^†^	*P *^‡^	OR (95% CI) ^†^	*P*^‡^	OR (95% CI) ^†^	*P*^‡^
Total	35	1.05 (0.95-1.16)	0.001	1.04 (0.96-1.12)	0.004	1.04 (0.97-1.13)	0.000	1.03 (0.96-1.11)	0.022

Ethnicities									

Asian	10	1.05 (0.93-1.20)	0.051	0.97 (0.88-1.07)	0.657	0.99 (0.90-1.08)	0.427	1.08 (0.92-1.28)	0.035

European	23	1.05 (0.92-1.19)	0.001	1.09 (0.99-1.20)	0.003	1.09 (0.98-1.21)	0.000	1.00 (0.94-1.07)	0.062

Source of controls									

Population-based	15	1.01 (0.92-1.11)	0.130	1.07 (0.95-1.21)	0.010	1.06 (094-1.20)	0.004	0.98 (0.90-1.07)	0.335

Hospital-based	20	1.07 (0.92-1.24)	0.000	1.02 (0.93-1.11)	0.048	1.03 (0.93-1.15)	0.002	1.06 (0.95-1.20)	0.014

Cancer types									

Lung cancer	9	1.08 (0.85-1.38)	0.017	0.99 (0.84-1.17)	0.059	1.03 (0.85-1.25)	0.007	1.04 (0.92-1.19)	0.086

Bladder cancer	7	0.96 (0.83-1.09)	0.312	1.01 (0.91-1.12)	0.376	0.99 (0.90-1.10)	0.453	0.95 (0.85-1.07)	0.360

Colorectal cancer	4	0.93 (0.55-1.57)	0.001	1.05 (0.78-1.41)	0.025	1.01 (0.72-1.43)	0.003	0.93 (0.63-1.39)	0.003

Breast cancer	4	1.20 (0.82-1.76)	0.042	1.19 (0.76-1.86)	0.000	1.23 (0.79-1.92)	0.000	1.06 (0.92-1.23)	0.345

Head and neck cancer	2	1.03 (0.80-1.32)	0.306	1.00 (0.81-1.23)	0.242	1.01 (0.83-1.23)	0.202	1.03 (0.83-1.27)	0.621

Others	9	1.07 (0.94-1.23)	0.156	1.05 (0.94-1.16)	0.754	1.05 (0.96-1.16)	0.609	1.05 (0.93-1.18)	0.111

* Number of comparisons

^† ^Random-effects model was used when P value for heterogeneity test <0.05; otherwise, fix-effects model was used

^‡ ^P value of Q-test for heterogeneity test

## Discussion

To data, numerous studies have been carried out to investigate whether polymorphisms in *APE1 *are associated with the risk of cancer; however, the data have yielded conflicting results. In the present study, to derive a more precise estimation of the relationship, we performed a meta-analysis of 37 published studies including 5,139 cases and 5,201 controls for *APE1 *−656 T > G polymorphism, and 14,222 cases and 19,746 controls for *APE1 *1349 T > G polymorphism. Our meta-analysis indicated that variant genotypes (GG and TG/GG) of −656 T > G polymorphism was associated with a significant decrease in the overall risk of cancer, especially for lung cancer and Asians. However, the 1349 T > G polymorphism did not appear to have significant influence on the overall risk of cancer.

Apurinic/apyrimidinic (AP) sites are common mutagenic and cytotoxic DNA lesions which caused by the loss of normal bases [49]. *APE1 *had been extensively studied as the major AP endonuclease involved in the repair of AP sites through both its endonuclease and phosphodiesterase activities [7]. Alteration in the expression of *APE1 *may influence its capacity to repair DNA damage. Several lines of evidence support that the −656 T > G polymorphism plays a role in influencing the promoter activity of *APE1*. Using in vitro promoter assay, Lo et al. found that the −656 G allele had a significantly higher transcriptional activity than that of the −656 T allele and individuals with the −656 G allele were at a decreased risk for lung cancer [13]. In their view, the "higher production" genotype for *APE1 *might offer protection against the development of lung cancer [13]. Nearly at the same time, Lu et al. also reported a similar protective effect of the −656 G allele against lung cancer risk in two independent studies [12]. However, they showed that the G allele attenuated the binding of Oct-1 to the promoter region of *APE1 *and resulted in decreased transcriptional activity. Given the disparity between these functional studies, the mechanism underlying the regulation of *APE1 *by this polymorphism may be still required further investigation. Nevertheless, our meta-analysis showed that individuals with the variant genotypes (GG or GG/TG) of −656 T > G polymorphism were associated with a decreased cancer risk than those with the TT genotype. Although publication bias was observed in the recessive genetic model, our meta-analysis with or without trim-and-fill method did not draw different conclusions, suggesting that our results were statistically robust.

In the subgroup analysis by cancer type, we found that individuals with the variant genotypes of −656 T > G polymorphism were associated with decreased lung cancer risk. However, the result should be interpreted with caution. Because, only four types of cancer (lung cancer, bladder cancer, brain tumor and colorectal adenoma) were included and the studies of each type were very limited, which may have insufficient power to reveal a reliable association. In the subgroup analysis by ethnicity, the results suggested that the association was more apparent among Asian populations. Several reasons may lead to the ethnic difference. First, differences in genetic background may cause the difference. Then, environmental or life style context which strongly vary between populations may play a role. Besides, other factors such as selection bias and different matching criteria may also result in the difference. In considering of the limited numbers of studies, in the future, large numbers of studies will be required to validate these associations.

Although it has been suggested the 1349 T > G polymorphism could influence the sensitivity to ionizing radiation [50], it does not result in reduced endonuclease activity [18]. A previous meta-analysis which investigated the association of 1349 T > G polymorphism and cancer risk suggested that the variant genotypes were associated with a moderately increased risk of all cancer types (OR = 1.09, 95% CI = 1.01-1.18 for TG versus TT; OR = 1.08, 95% CI = 1.00-1.18 for GG/TG versus TT) [51]. Compared with the previous study, we excluded four studies [46,52-54] in which the 1349 T > G genotype distributions in controls did not conform to HWE, since deviations from HWE in control subjects may bias the estimates of genetic effects in genetic association studies and meta-analysis. Besides, four additional studies with 2, 307 cases and 3, 184 control subjects in total were included in the present meta-analysis which could provide more solid evidence on the specific lack of association between *APE1 *1349 T > G polymorphism and cancer risk.. Given the power of this meta-analysis which included 14,740 cases and 20,533 controls, a false-negative finding is unlikely. However, further studies may be still needed to investigate the interactions between *APE1 *polymorphism and environmental factors which play important role in carcinogenesis but were not assessed in the present meta-analysis due to lack of original data. Besides, more attention drew to the prospective −656 T > G polymorphism in the further studies may be helpful to reveal the role of *APE1 *in the etiology of cancer.

Some limitations of our meta-analysis should be addressed. Firstly, the numbers of published studies collected in our analysis were not large enough for the comprehensive analysis, especially for the *APE1 *−656 T > G polymorphism. Secondly, lacking the original data of the included studies limited our study to further evaluate the potential interactions, since gene-environment and gene-gene interactions and even different polymorphic loci of the same gene may also modulate cancer risk. Besides, our results were based on unadjusted estimates, while a more precise analysis needs to be conducted if individual data such as age and sex are available. Nevertheless, advantages in our meta-analysis should also be acknowledged. First, a systematic review of the association of *APE1 *polymorphisms with cancer risk is statistically more powerful than any single study. Second, the studies included in our meta-analysis strictly and satisfactory met our selection criteria.

## Conclusions

In conclusion, the results from this meta-analysis suggest that the *APE1 −*656 T > G polymorphism, but not the *APE1 *1349 T > G polymorphism may contribute to genetic susceptibility to cancer. Further prospective studies with larger numbers of unbiased-matched homogeneous patients and well-matched controls are required to validate our results and to clarify the gene-gene and gene-environment interactions between *APE1 *polymorphisms and cancer risk.

## Competing interests

The authors declare that they have no competing interests.

## Authors' contributions

BZ carries out the meta-analysis study and drafted the manuscript. HS participates in the design of the study and performs the statistical analysis. YS and KX collect and extract the data. XS and WM have been involved in revising the manuscript critically for important intellectual content. QS conceives of the study, and participates in its design and coordination and helps to draft the manuscript. All authors read and approve the final manuscript.

## Pre-publication history

The pre-publication history for this paper can be accessed here:

http://www.biomedcentral.com/1471-2407/11/521/prepub
